# How Lifestyle Factors Affect Cognitive and Executive Function and the Ability to Learn in Children

**DOI:** 10.3390/nu11081953

**Published:** 2019-08-20

**Authors:** Jamie Jirout, Jennifer LoCasale-Crouch, Khara Turnbull, Yin Gu, Mayaris Cubides, Sarah Garzione, Tanya M. Evans, Arthur L. Weltman, Sibylle Kranz

**Affiliations:** 1 Center for Advanced Study of Teaching and Learning, Charlottesville, VA 22903, USA; 2 Department of Kinesiology, Curry School of Education and Human Development, University of Virginia, Charlottesville, VA 22903, USA

**Keywords:** child nutrition, diet, hunger, physical activity, learning, executive function, cognitive function, focus and concentration, diversity, socio-economic characteristics

## Abstract

In today’s research environment, children’s diet, physical activity, and other lifestyle factors are commonly studied in the context of health, independent of their effect on cognition and learning. Moreover, there is little overlap between the two literatures, although it is reasonable to expect that the lifestyle factors explored in the health-focused research are intertwined with cognition and learning processes. This thematic review provides an overview of knowledge connecting the selected lifestyle factors of diet, physical activity, and sleep hygiene to children’s cognition and learning. Research from studies of diet and nutrition, physical activity and fitness, sleep, and broader influences of cultural and socioeconomic factors related to health and learning, were summarized to offer examples of research that integrate lifestyle factors and cognition with learning. The literature review demonstrates that the associations and causal relationships between these factors are vastly understudied. As a result, current knowledge on predictors of optimal cognition and learning is incomplete, and likely lacks understanding of many critical facts and relationships, their interactions, and the nature of their relationships, such as there being mediating or confounding factors that could provide important knowledge to increase the efficacy of learning-focused interventions. This review provides information focused on studies in children. Although basic research in cells or animal studies are available and indicate a number of possible physiological pathways, inclusion of those data would distract from the fact that there is a significant gap in knowledge on lifestyle factors and optimal learning in children. In a climate where childcare and school feeding policies are continuously discussed, this thematic review aims to provide an impulse for discussion and a call for more holistic approaches to support child development.

## 1. Introduction

Learning, defined in this paper as acquiring new knowledge and skills, is a critical yet complex process in human development and is ubiquitous in early childhood. Young children learn everyday behaviors, skills, and other knowledge and functions (e.g., language) at a pace unparalleled by any other lifecycle stage. Both social skills and academic performance predict children’s probability to be gainfully employed later in life. Therefore, it is in society’s best interest that learning should be optimized for all children. 

**Figure 1:**
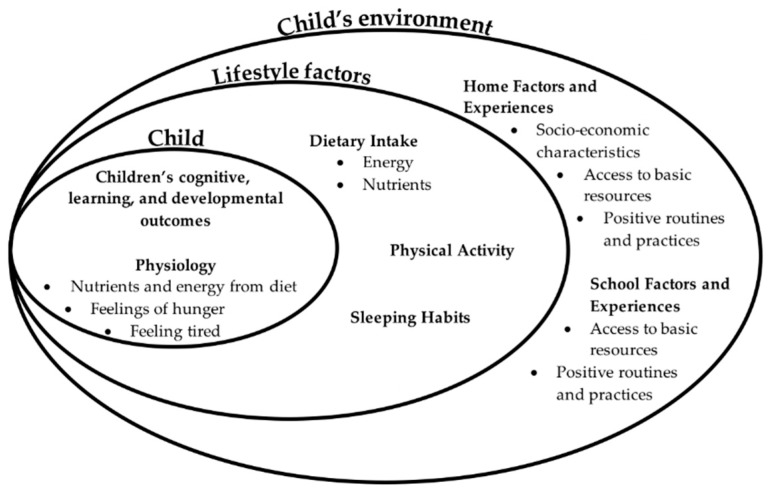
Contributors to children’s cognitive, learning, and other developmental outcomes are described in this ecological model. The inner-most circle represents the child’s own body, where physiological conditions, such as the nutrients and energy provided from meals and snacks, as well as the resulting feelings of hunger or tiredness, may directly influence the ability for cognitive processes. The lifestyle factors affecting the child’s physiology are dietary intake, physical activity, and sleeping habits. The outer-most circle represents the child’s larger environment, such as the home and the school. Socioeconomic factors have a substantial effect on access to basic resources, such as an area to play and be active, the noise level at bed time, and the familial established routines and practices. Likewise, the school’s resources, for instance playgrounds and green space, as well as routines and practices, affect the child’s lifestyle behaviors.

**Figure 2:**
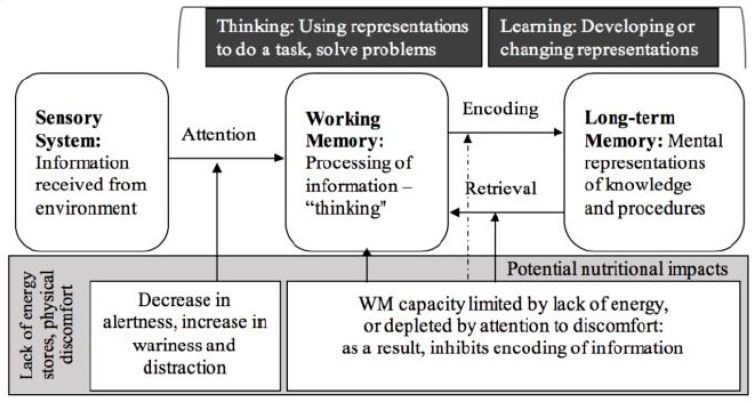
An example of the process involved in learning and potential impacts of nutrition, based on Information Processing Theory. The process of learning is a complex construct that can be described as a series of engagement in processing and information-storing/memory systems that ultimately result in knowledge. One of the most basic conditions for a child to be able to complete these tasks is having the necessary energy and no inhibiting factors of discomfort (e.g., hunger, fatigue). If conditions are not optimal, the child experiences lower levels of alertness, or wariness, thus decreases attention to any input from the environment. Thus, WM capacity will be restricted, limiting what information is eventually encoded and retained in long-term memory, from where it can be retrieved at a later time.

The foundational knowledge and skills acquired during early childhood set the trajectory for learning in the subsequent decades, if not for the entire life. The process of learning depends at least in part on the child’s physical well-being and sensations, such as discomfort, as we discuss below. There is a direct relationship between the modifiable lifestyle factors of diet, PA, and sleep on well-being. Furthermore, those three factors are greatly affected by the child’s social and economic environment. We generated visual models based on the combined research reviewed to explain those relationships in the larger environment (Figure 1, [1]) and the process of learning (Figure 2, [1,2,3]). If we understand learning as a system of cognitive processes (e.g., information processing model, [2]) and that these processes affected by diet, PA, and sleep (Figure 2), it becomes clear how influential these factors are for cognition and learning; we will discuss research on these lifestyle factors within the context of early childhood development. Furthermore, we will provide a call-to-action for future research to include modifiable lifestyle factors in the exploration of more effective interventions for optimal cognition and learning. 

The overall goal of this lead paper of the special issue on “Dietary Intake, Brain Development, and Learning,” is to evoke thoughts and to encourage fellow researchers, practitioners, and policy makers to consider modifiable lifestyle factors, such as diet, physical activity, and sleep. We will discuss the available evidence, the pathways by which these factors affect learning, and suggest potential application of current knowledge on educational practice, especially in childcare and school settings. 

### 1.1. Children’s Cognition and Learning

To date, cognitive and developmental literatures provide a wealth of knowledge about children’s learning and development, but very little is known regarding how to support children’s biological ability and readiness to learn through modification of lifestyle factors and the environment. Based on the well-established relationships between diet and physical functions, it stands to reason that diet and PA also affect cognitive function, or how well a person can learn. For example, using a simplistic definition of learning as changing existing mental representations of what someone knows or can do, Information Processing Theory would suggest that experiences and information (defined as external inputs to the brain) from the environment would first need to be attended to before children can process them in any way [2,3]. The ability to have/mindfully channel this attention is likely to be impacted by the child’s lifestyle, especially if the child is lacking adequate energy and feeling hungry. In order for a child to attend to something, working memory limitations modulate the availability of cognitive resources available to act on that information. Feelings of hunger likely absorb some of that limited attentional capacity [2,4]. In other words, diet is likely to influence children’s’ available working memory capacity to sufficiently process that information, to adequately encode information into long-term memory, and to recall information from long-term memory as needed (see Figure 2; these ideas are discussed in more detail later in the paper, and see [5] for this in the context of school nutrition). Based on this hypothesized interaction between physical sensations, reflecting nutritional status, and the effort needed for optimal information processing, academic learning outcomes (e.g., math and language skills) as well as conceptual understanding are very likely negatively influenced by suboptimal dietary intake. 

### 1.2. Diet and Nutrition

Diet and nutrition can be differentiated by two elemental functions: The energy that the human body can garner from the foods consumed and the nutrients that are supplied to the human physiological system. We address both of these functions in this review. While there is ample research in animal models to describe the relationship between individual nutrients and brain development and function, there is limited evidence on the effect of diet and nutrition on cognitive function in humans. Total diet intake patterns might be unfavorable for cognitive function in aging (e.g., the Western Diet has been implicated in accelerating the brain’s aging process), but diet can be beneficial (e.g., the Mediterranean Diet is associated with slowed aging of the brain and improved cognitive functioning [6]). 

Some studies indicate a relationship between individual diet constituents, such as omega-3 fatty acids, and brain function or learning in children [7]. The pathway by which this effect is asserted can be via direct contribution of the nutrient, such as for lutein or long-chain omega-3 unsaturated fatty acids, while other diet constituents function as intermediaries and modify a neural mechanism in the brain, in turn affecting cognitive function or learning processes [8]. Dietary fiber, which is present in many foods typical of the Mediterranean diet, potentially affects the relationship between diet and learning through two functions: (a) Dietary fiber directly contributes to blood glucose control and (b) dietary fiber is a critical component in the establishing and maintenance of the gut microbiome, the settlement of beneficial bacteria in the colon of the human digestive system. Neither of these two pathways have been studied in humans. The potential role of the “gut-brain-axis” in the human body has been hypothesized to be of critical importance; however, to date, only animal studies elucidate a possible direction and magnitude for this relationship [9]. 

### 1.3. Power Supply for Learning: Blood Glucose Availability

As explained in greater detail below, blood glucose levels play a major role in human cognitive processes. Blood glucose levels fluctuate in healthy humans in response to dietary intake. Glycemic index (GI) and glycemic load (GL) are terms we define in the next paragraph, as they are used to describe the effect of consumption of an individual food, beverage, or total diet pattern on the blood glucose response [10,11]. Blood glucose is the universal metabolic energy supply in the human system, and blood glucose levels rise within 30–45 min after ingestion of food. In healthy individuals, this increase of energy supply signals the release of insulin from the pancreas to activate the abundantly available cellular glucose uptake mechanism (GLUT-4), which triggers the removal of blood glucose into the cells of liver, muscle, and fat tissue, thereby reducing the blood glucose level [12]. A number of factors associated with metabolic control affect the GI and GL of foods in individuals, such as a person’s age or the food consumed simultaneously [13], or the macronutrient composition of a previous meal [14,15]; however, overall, GI and GL are good measures of carbohydrates in a person’s diet and energy that is available to the human body.

The GI and GL are related measures but they are not interchangeable. GI is an indication of the potential peak blood glucose level following food intake. This rise is predominantly based on simple sugars (mono- and di-saccharides), which are rapidly absorbed in the upper small intestine and enter the bloodstream in the gut lining to then be transported to the liver and the rest of the body via the vena cava. Complex carbohydrates, such as polysaccharides and starches, must first be digested in the gut lumen through the action of enzymes that cleave the large carbohydrate chains into absorbable, smaller units (mono- and di-saccharides). This process of digestion and absorption takes time, thereby delaying the release of food-based simple sugars into the blood, while some of the more rapidly absorbed carbohydrates have already been removed from the bloodstream into the cells. Since the GI of a food reflects the maximum increase of blood glucose levels, it is lower with increasing proportions of complex carbohydrates, such as dietary fiber, in the food, but it also depends on the preparation methods of the food [16]. Often, the GI of a food is compared to the glycemic response trigger by consumption of one slice of white bread (GI = 75) as a standard, or 100% of the response [11]; alternatively, a standardized load of 75 g of the mono-saccharide glucose is provided in liquid form during the oral glucose tolerance test (OGTT). In summary, the effect of dietary intake on blood glucose levels is well understood, and higher GI values indicate larger spikes in blood glucose level, signaling insulin release to clear the blood glucose from circulation [17]. Compared to the GI, the GL is a measure of diet-induced insulin demand and constitutes the GI of a food multiplied by the amount of that food (in grams) consumed [18,19]; thus, GL can be interpreted as the “glucose removal burden.”

In light of the current popular interest in low-carbohydrate diets, it is worth pointing out that not all foods with high amounts of carbohydrates have a high GI; for instance, barley (GI = 28) and corn tortillas (GI = 46) have relatively low GIs. Additionally, although it might be intuitive that all sugars should have a high GI, the monosaccharide glucose has the highest GI. with a value of approximately GI = 103. while the monosaccharide fructose has a low GI. with a value of approximately GI = 15 [11]. Ergo, a person consuming a food sweetened with fruit puree (fructose) has a very different glycemic response compared to consuming a food that is made with glucose (dextrose) or “table sugar,” which has an equal part of glucose and fructose. The difference between the GI of glucose and fructose is due to the very different metabolic pathway of the hexose (glucose) compared to the pentose (fructose). As this example shows, it is important to realize that policy on food programs can lead to very different physiological responses, depending on the food ingredients used. Further discussion of the metabolic pathways of mono- and di-saccharides is beyond the scope of this paper, but abundant information is available elsewhere [20]. One commonly pursued premise of consuming a diet high in sugars is that sweet diets induce increase appetite and food intake [21,22]. 

The biological need for energy supply is reflected in part through a complex endocrine system involving insulin fluctuations and individual’s feelings of hunger [23,24]. The indicators of hunger vary greatly between persons, and a variety of social-emotional factors contribute significantly to the sensation, frequency, and magnitude of feelings of hunger [25]. Studies in children indicate that diet variety (having higher numbers of different foods in the diet) is a significant predictor for the stimulation to eat and the types foods consumed [26], indicating that variety is one trigger to consume food, probably even in the absence of hunger. Furthermore, high obesity rates indicate that food intake is not limited to eating in response to biological need for energy, but has social-emotional triggers, such as eating due to stress, boredom, and other emotions; the act of eating itself, and some foods, have been hypothesized to be addictive [27,28]. As indicated above, feelings of hunger or feeling over-full both might affect children’s ability to process information in a manner conducive to learning. Eating in the absence of hunger, or overeating [29], is a contributor to excess body fat and childhood obesity [30]. Studies aimed at understanding the relationship between obesity and cognition during childhood have only recently emerged, and the majority of findings have been limited to the domain of executive control [31,32,33]. Our previous research has shown that children are able to report their feelings of hunger and that serving foods higher in fiber and protein lead to changes in the amount and variety of food consumed [34], thus, it stands to reason that a dietary change may also affect children’s ability to learn if frequency and duration of feeling hungry is reduced. 

### 1.4. Glucose in the Brain

The brain is a highly complex organ that begins developing in the embryo, and its plasticity extends throughout the lifespan [35]. Due to its complex structure and processing demands, the brain has high metabolic activity; although it only contributes approximately 2% of total body weight, it uses 20% of the total energy generated from food consumed [36]. Thus, dietary intake affects brain function in two ways: (a) The use of metabolic energy from different macronutrients (e.g., carbohydrates, proteins, and fats) and (b) the availability of individual micronutrients from foods (i.e., folate, iron, and iodine, which have important roles in brain function) [37,38]. 

The preferred source of energy in the brain is glucose [39], making the relationship between glucose intake and variables affecting the availability of glucose to the brain (e.g., PA, diseases or conditions affecting blood glucose control, fasting, or hyper-metabolism) especially important [40]. Since glucose cannot be stored in the brain [41], a steady blood glucose level is critical to ensure a steady energy supply; however, if glucose supply is limited, lactate or ketone bodies can be used for energy [42]. There is evidence for differential metabolism of glucose and ketones in the healthy young versus older adults [43], and the utilization of ketones as an alternative to glucose for energy in adults with mild cognitive impairment and Alzheimer’s disease [44]. However, the relationship between the use of these alternative energy sources and the ability to learn and cognitive function in children are not well understood to date. 

The connection between GI/GL and learning and cognitive performance is discussed below. For instance, differences in diet lead to variations in blood glucose level and energy utilization for processes regulated in the brain. High GI foods induce larger blood glucose fluctuations, which then impact several brain functions (e.g., short-term memory [45]). Furthermore, the GL of a meal determines how long blood glucose levels remain elevated [46]. The role of glucose on children’s cognitive ability and readiness to learn is confounded by the direct relationship between blood glucose and glucose availability to brain tissue. While cellular glucose uptake in the liver, fat, and muscle is regulated by the insulin-dependent GLUT-4 mechanism [47], the transporter mechanism into the brain (GLUT-1) is concentration dependent [48]. The relationship between blood glucose levels and cognitive functioning or learning is an area that remains unexplored. 

There has been an abundance of research on the role of breakfast. Overall, results suggest that the glycemic effect of breakfast can be influential in how much a student is able to focus and learn during a given school day [49,50,51]. This effect might be confounded by the well-described phenomenon of increased feelings of hunger in response to the consumption of high GI foods. Data indicate that high GI foods cause individuals to eat more [52], likely because the drop in blood glucose in reaction to insulin release, and the removal of glucose from the blood stream, triggers a strong hunger signal. Meta-analyses indicate that low GI but not low GL meals affect energy intake in children [53]. Overall, consuming a low GI meal results in increased satiety and decreased calorie consumption in subsequent meals, while high GI meals result in overeating and increased body fatness [10,53,54,55,56,57]. While not yet explicitly tested, one possible effect of experiencing hunger pangs after a high GI meal could be that children feel distracted by feelings of hunger. 

While some initial research in this area has emerged, much is still unknown about whether specific dietary changes can optimize children’s learning and cognitive processing. As discussed above, it is particularly important to recognize the role of GI and GL in foods served to children, in lieu of proportions of carbohydrates, fat, and protein in the foods and meals provided. 

Moreover, although dietary recommendations addressing the biological whole-body need for energy and nutrients are in place for childcare centers and schools, learning-focused food policies and practices are lacking [58]. In addition, the critical relationship between social disparity and access to a diet that supports learning is not well understood. Socioeconomic disparities and how they relate to neurological development and early cognitive abilities through nutrition emerge even before birth and show persistent effects [59,60]. The role of access to resources is discussed in another section of this review.

### 1.5. Nutrition and the Brain: Cognitive Processes and Learning

The impact of glucose on brain functioning generalizes to the cognitive processes involved in learning [61,62]. Although human brains include protective processes to maintain cognitive function across varying levels of nutritional status, for example, between meals, there is still some evidence of the impact of nutrition on cognitive processes involved in learning [61]. These include the more obvious impacts of nutritional deficits during critical developmental periods, but also show that daily nutritional consumption during childhood can influence cognition and learning. There is a good amount of research on the former, which we describe briefly in the areas of brain development and iron-deficiency anemia and in attention-deficit-hyperactivity-disorder. The latter, however, is a domain in need of much more research to provide the knowledge needed to inform educational interventions based on nutrition to support children’s learning. 

### 1.6. Nutritients and Brain Development

Some aspects of nutrition’s effect on cognitive development and performance are well studied and understood. For example, general malnutrition during prenatal development and the first months of life show life-long detrimental impacts on brain development that later manifest in a range of learning challenges (e.g., self-regulation difficulties and lower academic achievement) [60,63,64,65,66,67,68]. Research indicates that during childhood, iron has a large role, as infants suffering from iron deficiency show mental and motor impacts [69], and more general behavior that could impact learning being observed, such as weariness and fatigue [70,71,72,73,74,75,76]. Low iron status in slightly older children is linked to changes in neural morphology, myelination, and synaptogenesis during development [77]. During a shortage in the body, iron is preferentially directed (away from the brain) to the production of red blood cells. Thus, it is not surprising that numerous studies indicate that iron deficiency leads to impairments in both motor and cognitive function in children [78]. Importantly, the negative effects of iron-deficiency anemia are lasting [73,76,79,80] and negatively impact academic performance [81,82,83,84,85]. Despite so much evidence on the impact of nutrition for brain development, with effects shown to last over time, and the subsequent influence on learning, there is only limited evidence that nutrition-based interventions can have mediating effects on negative outcomes [72,86,87]. Thus, there is a need for nutritional interventions that examine the potential to support learning throughout development. 

Other micronutrients important for brain development and function are iodine, which has been shown to be critical for normal function of the brain, as demonstrated in a re-pletion study in individuals with iodine insufficiency [88]; and folate, as supplementation during pregnancy significantly decreases the manifestation of neural tube defects [89], and in school-aged children, folate [90] and vitamin B12 [91] intake has been associated with academic performance. Moreover, zinc is essential for normal brain development, playing an essential role in neuronal migration, neurogenesis, and synaptogenesis [92]. Although limited, there is some evidence that zinc supplementation results in improved attention and abstract reasoning [93]. Zinc deprivation has been linked to increased incidence of the neurodevelopmental disorder attention deficit hyperactivity disorder (ADHD) [94].

Greater levels of carotenoids, which are found in leafy vegetables, have been associated with higher scores on cognitive tests in the visual-spatial domain [95]. Lutein (L), one of the three major types of dietary carotenoids, is present in the brain [96], and metabolomics analyses indicate that it has “functional importance” on cognition and infant brain development [97]. The concentration of L is correlated with homocarnosine, a neuroprotective antioxidant found in the hippocampus and frontal cortex [97]. Interestingly, lutein concentration is higher in children than adults, suggesting a possible role in development [98]. L has been specifically related to the cognitive function measures of executive function, language, learning, and memory [98], and improved the speed of temporal processing in young adults [97]. It has also been associated with macular pigment density, which interacts with cognitive functioning [98]. 

Lack of protein results in a cascade of negative consequences at the brain level, including changes in protein phosphorylation, impaired neurotransmitter systems, decreased overall brain volume, and altered hippocampal formation [99]. Supplementation with protein in undernourished children has been shown to improve children’s cognitive performance, and is most effective in the first two years of life [100]. Children’s long-chain omega-3 fatty acid intake has been positively associated with relational memory [101], and adolescents’ fatty fish consumption has been associated with faster processing speeds compared to those consuming meats or a supplement [102]. Long-chain polyunsaturated fatty acid supplementation during gestation may improve crystallized intelligence in children [103] while saturated fatty acids’ intake is negatively related to memory [101].

Overall, research of individual nutrients indicates roles in cognitive function and the potential for learning. A variety of dietary factors during childhood may improve children’s ability to learn through a variety of mechanisms. However, well-controlled clinical trials in children to determine the strength of these relationships and potential thresholds of intake levels to yield beneficial results are lacking. 

## 2. Physical Activity (PA) and Fitness

There is an emerging body of evidence that supports a positive relationship between physical activity, fitness, cognitive function, and academic achievement [104,105,106,107,108,109,110], although less information is available on the interaction between physical activity and nutrition in increasing cognitive function and/or academic achievement [111]. The terms used in this section are defined to increase accuracy and understanding [105]. 

Increasing physical activity does not necessarily increase fitness, which is commonly defined as “The ability to complete the tasks of daily living without undue fatigue” [105]. Increased fitness is associated with improved athletic performance and improved health. Components of fitness include cardio-respiratory endurance, muscular strength and endurance, flexibility, and body composition. Exercise is “A subset of PA that is structured, repetitive, and designed to improve or maintain fitness as a primary objective.” In the sections that follow, we identify the systematic reviews and metaanalyses that support the role of physical activity and/or increased fitness for improving cognitive function and provide some examples of more recent research findings.

### 2.1. Physical Activity for Enhancing Cognition and Learning in Children

There are a number of systematic reviews and meta-analyses that describe the benefits of physical activity, classroom-based physical activity, and activity breaks (e.g., recess) on cognitive function and academic outcomes [104,105,106,107,110], although there are few high-quality studies that have assessed the effects of physical activity interventions within the school setting on executive functions.

Aadland and colleagues recently examined the effects of a seven-month curriculum prescribed PA intervention (the Active Smarter Kids (ASK) intervention) on executive function in 10-year-old Norwegian children [112]. They examined data from 57 schools (from the original 60 recruited), resulting in 1129 (48% female) 10-year old participants. All schools participated in the curriculum-prescribed 90/min per week of physical education and 45 min/week of PA (total of 135 min/week). The ASK intervention schools (*n* = 28) generated an additional 165 min per week of PA by implementing: (1) Physically active educational lessons (3 × 30 min/week) in the subjects of Norwegian, mathematics, and English; (2) PA breaks during classroom lessons (5 min/school day); and (3) PA homework (10 min/school day). The PA educational lessons and the PA homework also incorporated learning tasks in the PA, adding cognitive load to the activity. Several tests of executive functions were assessed, including inhibition, working memory, and cognitive flexibility by four pen and paper tests. Inhibition was assessed using the StroopCW test; a semantic verbal fluency test was used to assess cognitive flexibility; and the Digit Span test from the WISC-IV was used to assess working memory.

Although the initial analyses did not reveal an effect of the ASK intervention on executive functions, almost one-half of the control schools reported more PA than prescribed. When this was controlled for in subsequent analyses, there were significant ASK intervention effects on the composite score of executive functions and cognitive flexibility.

It should be noted that the ASK intervention did not result in improved aerobic fitness, and the total daily PA equaled ~60 min per day [112]. The latter may explain why the improvement in executive functions in the ASK study [112] were less than what has been observed in previous studies, where a dose-response relationship between PA and executive functions was observed (e.g., higher intensity and/or volume of PA is associated with greater improvement in executive function) [113,114]. As not all PA programs improve fitness, it is important to examine the independent effects of increased PA and improved fitness for enhancing cognition and learning in children.

### 2.2. Improved Aerobic Fitness for Enhancing Cognition and Learning in Children

Aerobic fitness has also been associated with enhanced cognition and learning in children [104,105,106], and since a dose response relationship between improved fitness and cognition and learning in children has been reported [113,114], improving fitness through PA may be more effective than focusing on increasing PA. This can start early in life, as the feasibility of integrating PA in young children attending preschools has been established [115]. 

There are several mechanisms relating increased fitness with changes in brain structure that could enhance cognition and learning in children. Cross-sectional data suggest that fitness may be associated with differences in the volume of specific regions of the basal ganglia [104], a brain region known to support aspects of procedural learning [116,117]. Lower-fitness children exhibit decreased inhibitory control, coupled with a smaller dorsal striatum, whereas aerobic fitness is associated with an increase in volume of the globus pallidus [104]. Additionally, volume in the hippocampus, a well-established hub of the brain’s memory network [118,119] has also been related to aerobic fitness [104] and PA [120]. It has been suggested that increased aerobic fitness might enhance cognitive development in children by changing volumes of specific brain regions involved in cognitive function [104].

Aerobic fitness also affects white matter volume in children. Data from the ActiveBrain project and the FITKids project indicate that aerobic (cardiorespiratory) fitness was associated with greater white matter volume in the inferior fronto-occular, inferior temporal, cingulate, middle occipital, and fusiform gyri in overweight and obese children. In the ActiveBrains project, motor fitness was related to increased white matter volume in six regions and increased muscular fitness was associated with increased white matter volume in two regions. The white matter volume of six of these regions were related to academic performance. The authors speculate that increased cardiorespiratory and muscle fitness may positively influence white matter volume, and in turn, academic performance [121].

More recently, Chaddock-Heyman and associates [122] reported that aerobic fitness was associated with greater cerebral blood flow, a metric of brain function, in children. These authors found that higher levels of aerobic fitness were associated with increased hippocampal blood flow in 7- to 9-year-old children independent of age, sex, and hippocampal volume. They went on to suggest that aerobic fitness may influence the plasticity of the developing brain, and that fitness may influence the metabolic demands of the brain in a region important for learning and memory, via enhanced blood flow. As PA is decreasing in children, and as more and more children are becoming increasingly unfit and sedentary, the authors suggest that their findings have important implications for learning [122].

### 2.3. Role of Exercise Intensity

It is well established that intensity of exercise is the most important exercise prescription parameter for improving fitness [123]. Several recent studies suggest that exercise intensity may be key for improving cognitive function, learning, and academic achievement in children and adolescents.

Moreau et al. reported that high-intensity training enhances executive function in children in a randomized placebo controlled trial [124]. The authors tested the effect of high-intensity training (HIT) compared to an active control on measures of cognitive control and working memory in children aged 7–13 years. Results indicated that the 6-week HIT program resulted in improvement on measures of cognitive control and working memory. However, this study only compared HIT to active controls, and as such, the effects of exercise intensity per se were not examined.

Jeon and Ha [125] examined the effects of three differing intensities of exercise on brain-derived neurotropic factor (BDNF), insulin-like growth factor 1 (IGF-1), and working memory, in 40 male middle school students. BDNF production has been shown to induce hippocampal neurogenesis and improve working memory, and is thought to be a primary factor in neurogenesis and neuroplasticity. IGF-1 is related to neurogenesis, regulation of the BDNF gene, and is involved in neuronal growth and differentiation. The authors randomly assigned subjects to one of four groups: Low, moderate, high intensity exercise training, or a stretching control group. Exercise training was performed 4 × per week for 12 weeks and clamped at 200 kcal per session. Their results revealed that the exercise training increased BDNF, IGF-1, and working memory in an intensity dependent manner. The authors suggested that there may also be an interaction between BDNF genotype and exercise, as met^66^ carriers showed larger gains in cognitive control and working memory than val^66^ homozygotes [124].

Most recently, Pindus and colleagues [108] examined daily PA patterns in children between the ages of 8 and 10 (49% girls). The subjects wore an Actigraph wGT3X+ accelerometer on the hip for 7 days. Moderate (MPA), moderate-vigorous (MVPA) and vigorous (VPA) epochs of daily PA were assessed. The investigators related intensity of PA patterns to brain function. A strength of this study is that PA was measured with shorter epochs compared to previous studies of PA. Although the authors did not observe any significant associations among daily VPA or MVPA on either behavioral or neuroelectric indices of cognitive control, exploratory analyses indicated that a specific accumulation pattern of time spent in an individual VPA epoch may be related to optimal brain function. VPA bouts lasting 30 s or longer had the most consistent associations with efficiency in cognitive processing across epochs and task conditions that varied in cognitive demands. The authors suggested that rather than total daily VPA, bouted VPA (e.g., bouts of 30 s or longer) may result in the greatest cognitive control in preadolescents.

### 2.4. Interaction between Exercise Intensity and Cognitively Engaging Activities

Although higher intensity exercise may be associated with improved aspects cognitive function, there are data that suggest that a combination of PA and cognitively engaging activities during PA may result in a more pronounced effect [109]. Egger et al. examined the effects of three different 20-week classroom-based PA programs on cognitive outcomes in children aged 7–9 years [109]. Children were assigned to high physical exertion and high cognitive engagement, high physical exertion and low cognitive engagement, or low physical exertion and high cognitive engagement groups. Executive functions (updating, inhibition, and shifting) and academic achievement (mathematics, spelling, and reading) were measured pre- and post-intervention. Results indicated that only a combination of long-term PA breaks with high cognitive engagement led to a stronger improvement in executive functions, with the relationship between shifting and academic achievement supported.

### 2.5. The Interaction between Nutrition and Physical Activity for Enhancing Cognition and Learning in Children

Although both nutrition and physical activity have been associated with enhanced cognition and learning in children, few data have examined the independent and combined effects nutrition and PA have on those outcomes. A recent study examined the seventh wave of the Early Childhood Longitudinal Study, Kindergarten Class 1998–99 (ECLS-K) dataset [111]. Data from the final wave, the eighth-grade year, were analyzed, resulting in a sample size of *n* = 9720. Scales were developed to classify subjects as having healthy or unhealthy nutrition, and as being physically active or non-active. Linear regression analyses were used to examine the relationship among nutrition, PA, and academic achievement, while controlling for socioeconomic status (SES), age, and sex. Results indicated that PA, nutrition, and the interaction of PA and nutrition were all significant predictors of reading, math, and science scores, while controlling for SES, age, and sex. Subjects classified as non-active, unhealthy nutrition, scored lower for reading, math, and science standardized tests when compared to subjects classified as active, healthy nutrition. These data highlight the need for prospective studies that examine the additive or surpra-additive effects of appropriate PA combined with a healthy diet on cognitive and learning outcomes in children and adolescents.

The data reviewed above suggest that PA/exercise of sufficient intensity and appropriate epochs with cognitive engagement and appropriate nutrition may be necessary to induce optimal cognitive improvements in children and adolescents. There is still much to learn about the interaction and optimization of these factors for improving cognitive outcomes. As childhood and adolescence is a period of important neurogenesis, identifying the optimal combination of the aforementioned factors, diet, PA, and sleep hygiene may be critical to improve memory and academic achievement.

## 3. The Role of Sleep on Cognition, Learning, and Development

As with diet and PA, children’s sleep health—as indicated by the duration, timing, and quality of sleep, and the presence of sleep disorders—is an additional modifiable aspect of children’s functioning, with critical connections to children’s learning and development. Sleep health is also directly related to children’s diets [126]. Research has revealed a vital role for sleep in physical and mental health and wellbeing, including memory formation and learning [127], cognitive function [128], expressive and receptive language [129], and social and emotional function [130]. For example, shorter sleep duration is associated with externalizing behavioral problems and lower cognitive performance at school entry [131]. As another example, longer sleep onset latencies, multiple night disturbances, and the presence of insomnia are associated with lower child IQ measures, and problems with self-regulatory abilities, such as following instructions [132].

Critically, the effects of compromised sleep on cognitive functioning and achievement may be greater for children from lower-SES backgrounds than for more advantaged children [133]. Children of low-SES backgrounds exhibit poorer sleep health than their more advantaged peers [134], which may help explain variability in school achievement for children from lower- and higher-SES backgrounds. For example, children from lower-SES families exhibit more parent-reported sleep issues, shorter sleep durations, and greater variability in sleep onset than children from higher-income families [135]. Possible mechanisms explaining these linkages include increased pre-sleep arousal due to worries or daytime stressors, and physical environmental conditions that may interrupt sleep [136]. Moreover, lower-SES households are less likely to meet pediatric sleep practice recommendations, (e.g., age appropriate bedtimes and wake times; meeting a child’s needs during day), than higher-income households [137]. For example, young children from disadvantaged households are less likely to have a consistent bedtime or bedtime routine than their more advantaged peers, which may contribute to decrements in sleep quality, and ultimately to children’s behavioral, cognitive, and health outcomes [138]. Children from low-SES households also experience more chaos in the home, including greater levels of crowding, noise, school and residential relocation, and parental partner instability [139], compared to their more advantaged peers—all of which may pose challenges to sleep health. 

Sleep health appears to be deeply intertwined with children’s diet and nutrition, and physical activity, in relation to child cognition, learning, and development. For example, short sleep duration is associated with higher markers of obesity in adolescents, with the association accounting for a combination of increased food intake and more sedentary habits [140]. As another example, a systematic review and meta-analysis of interventions targeting sleep and their impact on children’s BMIs, diets, and PA revealed that, of the included studies reporting improvements in children’s sleep duration, a positive impact on child BMI, nutrition, and physical activity was also observed [141]. Comprehensive research examining children’s sleep health, diet and nutrition, and physical activity, would represent a significant and innovative focus for prevention and intervention and yield a more complete understanding of how these factors interact to both support and reduce inequalities in child learning and development. 

## 4. The Role of Family Socioeconomic Status in Children’s Nutrition and Learning: Examining the Pathways

### 4.1. Considering Socio-Economic Status in Relation to Children’s Nutrition and Learning

Children’s health, wellbeing, and development are critical contributors to both concurrent and future functioning, including educational attainment [142,143,144,145], health [142,143,146,147], and overall quality of life [144,145,146,148,149]. Suboptimal nutrition and inadequate opportunities for early learning, both of which factors are inversely linked to socioeconomic status (SES), pose specific and significant risks to poor health, wellbeing, and developmental outcomes [77,150,151,152]. SES refers to a complex, multidimensional construct that reflects financial resources and capital, with the most common indicators of SES being household income, parental education, and parental occupation [153,154,155]. Because SES has documented links to nearly every imaginable aspect of child development, considering its role in child learning and nutrition is critical. Moreover, recent empirical studies reveal a significant, negative association between nutrition intake and learning among children from low-income families. Specifically, disadvantaged children with less healthy diets demonstrate lower math skills at kindergarten entry [156]. Thus, examining the potential pathways through which SES can impact learning and nutrition is warranted.

The role of SES, and low SES in particular, touches many children’s lives. The Lancet estimated nearly a decade ago that more than 200 million young children living in developing countries were not reaching their full developmental potential due to limited access to necessary nutrition, care, and education [157]. Since that time, the United Nations International Children’s Emergency Fund (UNICEF, New York, NY, USA) has estimated that, in developed countries, one in five children live in poverty (i.e., household income <60% of the country median), with 12.7% of children experiencing food insecurity and 31.4% of children not achieving baseline educational standards for their country [158]. In the US specifically, one of the wealthiest nations in the world, nearly one in five children experiences food insecurity and one in three fails to attain minimum proficiency in reading, mathematics, and science at school entry [159].

Although SES is a complex and multifaceted construct, it is also simplistic in that it may obscure critical mediating processes, modifiable factors, and key potential correlate of child development. Thus, this section begins by exploring more deeply how young children around the world are faring in regard to nutrition and learning as a function of SES. The relationships between economic resources and access to diet and opportunities for PA and the development of good sleep hygiene are well established. The sections that follow elucidate multiple potential pathways through which SES relates to child nutrition and learning and development, including access to basic resources, home experiences, and early care and education experiences. 

### 4.2. How Are Young Children Around the World Faring with Regard to Nutrition and Learning?

In 2018, UNICEF and the World Health Organization (WHO, Geneva, Switzerland), along with the World Bank, estimated 150.8 million children under age 5, worldwide (22.2%), to be stunted (i.e., too short for one’s age), 50.5 million (7.5%) to be wasted (too thin for one’s height), and 38.3 million (5.6%) to be overweight (i.e., too heavy for one’s height) in 2017. Importantly, large disparities exist between developed and developing countries concerning proportions of young children suffering from malnutrition. Specifically, 91% of all stunted children and 92% of all wasted children lived in lower-middle- and low-income countries. About one-third of children in developing countries (31.5% in lower-middle-income and 35.2% in low-income countries) were stunted, as compared to fewer than 7% of children in developed countries (6.9% in upper-middle-income and 6.1% in high-income countries). About one child in ten in developing countries (11.5% in lower-middle-income and 7.4% in low-income countries) suffered from wasting, as compared to fewer than 2% of children in developed countries (1.9% in upper-middle-income and 0.7% in high-income countries). Concerning child overweight status, percentages of overweight children in developed and developing countries did not differ significantly, though the number of overweight children has increased significantly more in lower-middle- and low-income countries (2.7 and 0.8 million children, respectively) than upper-middle- and high-income countries (0.5 and 0.4 million children, respectively) since 2000 [160]. Similarly, prevalence rates of malnutrition deficiencies in micronutrients (i.e., vitamins and minerals), are greater in developing countries than developed countries. For example, vitamin-A deficiency affects one third of children in developing countries where zinc deficiency is endemic as well [161]. 

In the US, approximately 2% of children under age 5 years were stunted, 6% were overweight, and 1% were wasted in 2012. Additionally, 13% had Vitamin-A deficiency, and 24% had iodine deficiency in 2012 [162]. More recently, in 2017, the National Center for Health Statistics (NCHS, Hyattsville, MD, USA) reported 13.9% of preschool-aged children to be obese in 2015 to 2016 [163]. Income- and education-based gaps are prevalent in American children’s health status. For example, Braveman and colleagues (2010) reported the percentage of children ages 0–17 years living in a household below 100% of the federal poverty line (FPL) with non-excellent or not-very-good health status tripled that of their peers living in a household above 400% of the FPL. The percentage of children with limited activity due to chronic disease among those who live in household below 100%-FPL is ten times as many as those who live in household above 400%-FPL. In addition, children in disadvantaged households exhibit lower scores on a healthy eating index compared to their peers from more advantaged households. Similar discrepancies exist between children living with a head of the household with lower than a high school degree and their peers living with a head of household with a college or graduate degree. Further, the United Nations Educational, Scientific and Cultural Organization (UNESCO, Paris, France) estimated more than one-half of all children (387 million) are not achieving minimum learning proficiency levels by the end of their primary education years; of these children, 39% are from the developed countries. Only one child in four, and one child in ten in lower-middle-income countries, will attain the minimum proficiency level in reading and mathematics [164].

In the US, more than 30% of children in Grade 2 or 3 failed to achieve at least a minimum proficiency level in reading, and about 5.3% failed in mathematics in 2015 [165]. However, learning gaps are evident even before children’s entry to formal schooling. For example, using nationally-representative sample of children from the 2010 kindergarten cohort of the Early Childhood Longitudinal Study (ECLS-K), Latham [166] reported one-half of all children had low proficiency in language, literacy, cognition, and general knowledge at kindergarten entry. In addition, 22.8% of children had poor approaches to learning and 16.7% had poor self-control. Large gaps existed in learning between young children from lower-income families and their peers from higher-income families. For instance, children from lower-income families performed an average of 1.26 standard deviations (SD) lower in language and literacy skills, 0.8 SD lower on a working-memory task, and 0.5 SD lower on a cognitive-flexibility task compared to their peers from higher-income families. Similarly, children from lower-income families were far more likely to demonstrate poor self-control abilities and poor approaches to learning [166].

### 4.3. Potential Pathways: SES and Access to Basic Resources

Although the causal effect of SES on child development has not been estimated, in social science, two central pathways have been identified through which SES may influence children’s development [167]. The first is the most direct and concerns home and neighborhood resources. At the home level, examples of resources include housing quality, nutritious foods, and health insurance. At the community level, resources include safety, food access, and other physical characteristics of the community. For example, children from poorer households are far less likely than their peers from more advantaged households to have books at home, to be engaged in early learning, to attend an early-childhood-education program, and to access adequate care [168].

Low quality of housing and food insecurity are two factors that socioeconomically disadvantaged children face every day. Besides having less housing affordability, low-income families live in houses with more structural defects, more exposure to toxic substances, less access to safe drinking water, and more overcrowding, when they are compared with their high-income counterparts [169]. Food insecurity is associated with being overweight, obesity, and undernutrition. According to the Food and Agricultural Organization of the United Nations’ 2018 report, these forms of malnutrition coexist (a phenomenon known as the “double burden” of malnutrition), are more prevalent in low- and middle-income countries, and are concentrated among poorer populations within countries. In the United States, Mexican–American children, children from households below the poverty thresholds, and children living in poor neighborhoods are more likely to experience food insecurity [170,171].

At the community level, low-income neighborhoods are more likely to be physically hazardous for children and lack basic infrastructure to support health development [169]. Further, an emergence of food deserts, defined as “regions in which access to food retailers that stock fresh, affordable, and healthy food options are lacking or nonexistent,” [172] has emerged [173]. In the United States, predominantly white neighborhoods have more supermarkets, whereas low-income urban areas have a higher number of smaller stores that charge higher prices and offer lower quality food [174,175]. The built environment is another characteristic of the neighborhood that has been analyzed by its potential to facilitate physical activity. The SES gradient is repeated, such that low-income, less educated, and predominantly minority neighborhoods have fewer parks and other recreational facilities [176].

### 4.4. Potential Pathways: SES and Children’s Home Experiences

Beyond access to basic resources, another pathway linking family SES with nutrition and child learning is the quality of home experiences. Prior research linking family SES and associated home experiences with diet and nutrition has examined a number of constructs, such as family routines, limit setting, household chaos and crowding, and the home environment more broadly [177]. For example, as compared to preschool-aged children from higher-SES backgrounds, children from lower-SES families are more likely to live in an ‘obesogenic’ home environment in terms of nutrition and feeding practices (e.g., availability of sugar-sweetened drinks), physical activity (e.g., lack of parent support for child physical activity), and media/TV use (e.g., TV in child’s bedroom; [178]). Additionally, for children from lower-SES backgrounds, eating recommended amounts of vegetables daily is more likely when children have family dinner seven days per week, eat a carryout meal no more than one day per week, eat a home cooked meal six or more days per week, or cook together with a caregiver five or more days per week [179]. Higher levels of family routines are also positively associated with indicators of children’s school readiness. For example, participation in family meal times is positively associated with preschoolers’ social and emotional competencies [180]. As another example, higher levels of family routines in preschool are associated with declines in teacher-reported behavioral problems and gains in prosocial behaviors from preschool to kindergarten, as well as greater gains in reading and math scores, and greater improvements in physical health over the same time period [181]. It is likely that predictable family routines such as mealtimes, bedtimes, and physical activity serve as markers of household organization, and may prepare and support children as they transition to formal schooling [180,181].

Further, linkages between family SES, child home experiences, and learning have been well-established in the empirical literature [182,183]. For example, access to learning materials within the home, coupled with parents’ efforts to involve children in stimulating and enriching experiences, are related to children’s school competencies in mathematics [184], language and literacy [185], early motor development, and social and behavioral development [182], to name but a few areas. Theory also suggests that children’s learning is a function of numerous multi-faceted and interrelated home experiential processes. As illustrated by the Family Stress Model, family SES is negatively associated with child learning and adjustment via pathways of family stress and conflict [186,187]. Specifically, households experiencing economic hardships and pressures also exhibit greater levels of stress, increased family relationship conflict, lower quality parent-child interactions, and decrements in child cognitive outcomes and social and behavioral competencies. As with access to basic resources, a growing body of evidence and theory indicates familial SES and associated home experiences may play a central role in child learning through the pathway of child diet and nutrition.

### 4.5. Potential Pathways: SES and Children’s Early Care and Education Experiences

Another pathway linking familial SES with nutrition and learning is access to and quality of early childhood care and educational experiences. Effects of early childhood care and education on child development and learning are well established in the field [188]. Consistent evidence points to high quality programs having impacts on language, literacy, cognition, and social-emotional skills [188,189,190,191]. High-quality early childhood care and education is also positively associated with child health and wellbeing, including healthy eating habits and sufficient levels of physical activity [192]. High-quality early care and education is also associated with advantageous later-life outcomes, including lower levels of poverty, increased participation in the work force, and improved health [193,194,195]; and may be particularly impactful for children from at-risk backgrounds, such as low-SES households [193,196].

Nutrition interventions have a long history of positively impacting children’s immediate health [197] but only recently have other outcomes have been investigated. In a recent US study, adults whose family received governmental supplemental nutrition assistance (SNAP) as children (compared to adults who did not) had a significantly increased rate of high school completion, and decreases incidences of stunted growth, heart disease, and obesity (SNAP Fiscal year 2014). More recently, efforts have been made to examine how early care and education interventions address cognitive development. Interventions focused on development were consistently found to promote children’s development, whereas nutrition interventions were found to benefit children’s growth outcomes and sometimes benefit children’s development [198]. In Aboud and Yousafzai’s [197] recent review of interventions since 2000, psychosocial-stimulation interventions exhibited a medium-effect (d = 0.43) on children’s cognitive development and nutrition supplementation; and education interventions showed a small-effect size (d = 0.09). Those findings suggest that perhaps integrating nutrition with early child development interventions may provide the most benefits for children’s development.

Due to the growing evidence base of early childhood intervention impacts, many countries have increased their investment in early childhood care and education programs as a way to help disadvantaged young children catch up [199,200]. Thus, universal or quasi-universal access to at least one year of early childhood care or education is now available in Organization for Economic Cooperation and Development (OECD, Paris, France) countries, with more than 90% of children enrolled in education at age 5. Disparities, however, exist. In low and middle-income countries, schooling has increased, but it has not been accompanied by increases in quality, and gaps in completion rates in all educational levels persist between SES and genders [172,201,202]. In the US, during the last decades, the early learning opportunities for low-income children have increased through public-funded pre-kindergarten programs that have been characterized by their high quality [203]. At the same time, the US has lower preschool participation rates than 70% of the member countries of the OECD, and the attendance is higher for children whose parents’ education is equal to college or more [204,205]. Thus, children who need it the most are less likely to have access [199,206] and more likely to be in a low-quality program if they attend [207,208,209,210,211,212].

## 5. Application and Practice Example

### 5.1. School Nutrition, Cognition and Learning

As discussed above, federal feeding programs play a critical role in children’s long-term development. Furthermore, many low-income children depend on the nutrition provided during childcare and school. These opportunities to provide nutrition to the majority of American youth has been extensively studied, with very conflicting results. 

School feedings include breakfast, snacks, and lunch, but do not include competing food sources, such as vending machines, bake sales, and other cash-based options. Research on the importance of breakfast, albeit controversial, continues its coining as the “most important meal of the day.” Some consider breakfast a fundamental component of the school day to support learning potential [213]. Research on breakfast includes both experimental studies of the short-term effects of breakfast eating, and longer-term effects studied through survey research. Within the same students, skipping breakfast showed negative influences on both memory and alertness, which could have adverse effects on academic learning [214]. Students who skip breakfast have lower school performance, and this relationship is partially mediated by attention [215]. Other studies found negative associations between skipping breakfast and memory and attention [5,216,217]. Attention and memory were also found to improve with a school breakfast program [218]. 

Survey studies of habitual breakfast eating on longer-term school outcomes show negative impacts for learning with habitual breakfast skipping [219], especially for math [220]. Positive learning benefits are associated with eating breakfast more often [221] and are observed over time in schools that have breakfast programs [222,223,224]. More generally, a meta-analytic review of high-quality studies of breakfast consumption versus no breakfast showed positive effects from eating breakfast, especially for highly demanding tasks and for children who are considered more vulnerable (e.g., lower SES, lower IQ, lower health, and poorer diets) [5,225]. Despite these findings, there is also reason to be cautious in the interpretation of the research on breakfast consumption [226]. Several of the studies mentioned above, showing the effects of breakfast on some measures, also included other measures of cognitive ability that were not affected by breakfast consumption. Still other studies found no associations between breakfast consumption and any measures included, or even negative effects of breakfast—perhaps due to the type of foods consumed or the extra, unneeded calories if children had a breakfast at home before school [5]. Yet, overall, Hoyland and colleagues’ [5] interpretation of their meta-analysis indicates positive effects. 

There are likely many reasons why breakfast is found to be important for school learning beyond general contribution to needed energy, such as reducing absenteeism [224,227,228]. For this review, these studies on breakfast serve as examples for effective, timely means to deliver important micronutrients and macronutrients needed for cognitive processes involved in learning. There is some evidence to support that the nutritional content or quality of breakfast matters for the positive outcomes observed [229,230]. Yet, many of the breakfast studies do not provide detailed information or analysis of the nutritional value of the meals. The importance of considering the type of food served for breakfast is exemplified in a study conducted with 290 children, which showed that those children with habitual rice-based breakfasts (as compared to bread-based breakfasts) had significantly larger gray matter volume (percent of gray matter volume per intercranial volume) in the left superior gyrus, while the bread group had significantly larger gray and white matter volumes in right frontal-parietal cortex, as measured using magnetic resonance imaging (MRI) [49]. Although both of these breakfast types might have been superficially described as “carbohydrate-based,” the measurable difference in outcomes demonstrates the importance of conducting detailed analysis of the types of foods consumed. It is noteworthy to point out that although the two breakfast types in this study might have been similar enough to create similar results, a study of dietary intake patterns, often termed “usual intake patterns,” might result in very different nutrient profiles. 

As discussed earlier, it is likely that nutrition can influence cognition and learning in several possible ways. When identifying possible nutritional mechanisms, it is also important to understand the specific cognitive and learning-related processes being impacted that relate to learning rather than focusing on overall academic performance. Despite the idea of children being curious, motivated learners who soak up new information, their cognitive processing is similarly complex as adults, with several ways that learning can be negatively impacted. For example, as we show in Figure 2, children’s initial attention to information during learning experiences can be impacted by their diet-related attention or weariness, either through their energy level or their motivation. Nutritional effects on learning-related behaviors have been found for level of activity and exploration [231], as well as arousal and motivation [232]. If children do attend to the environmental input, their feelings of hunger or lack of energy can still be a distraction, usurping working memory resources needed to process information and think. This can cause children to miss or misunderstand pieces of information, or limit their learning to shallow connections and memories, making it more challenging to remember and use the information learned; for example, by storing isolated content rather than constructively building on prior knowledge. 

The complex process of learning is cognitively demanding, even for young children. Yet, early education is often made effective by being situated in playful experiences, a powerful pedagogical approach [233]. When instruction is designed to foster intellectual engagement and curiosity, nutrition can detrimentally affect learning by causing unresponsiveness [234]. In fact, early nutritional deficiencies have even been found to cause later reduction in curiosity [235]. With the importance of curiosity in education [236], this could be an important factor to explore in relation to the influence nutrition might have on children’s learning. Nutrition can also influence older children’s learning by impacting cognition, as well as learning-related perceptions, such as self-esteem [237]. When researching to better understand how to support cognition and learning, nutrition should be an important consideration to maximize the effectiveness of interventions, and more research is needed to understand what it means to influence cognition through nutrition. 

Nutritional health of children, especially adequate levels of nutrients like iron and a healthy diet more generally, positively correlates to a range of cognitive performance measures [213]. Some experimental work has found that supplementing children’s nutrition with fortified food leads to cognitive benefits. For example, van Stuijvenverg and colleagues provided 6–12 year-old children with fortified snacks (iron, iodine, and beta-carotene) over a full year and observed positive effects on working memory, as well as better school attendance, because of decreased illness—which would lead to more learning experiences [238]. Attention, abstract and conceptual reasoning, and motor skills benefitted from a zinc treatment with other select micronutrients in a large sample of 6–9 year old children [238], and similar non-verbal intelligence benefits were observed for children ages 12–13 after only a three-day supplement of vitamins and minerals [239] and 12–15 year-old children after 12-week supplementation [240], though other studies show more mixed findings [241]. 

### 5.2. Specific Nutritional Components and Specific Cognitive Processes

Nutritional effects on behavior have been found for level of activity and exploration [231], as well as arousal and motivation [232]. While this work and others suggests the importance of adequate energy from nutrition to support learning and cognition, there is often the belief that the specific nutritional content is important—for example there is a largely held theory that sugary diets cause hyperactivity. Though there is some early correlational work that showed some support for an association [242], the overall results have been mixed [243]; experimental studies somewhat consistently do not show a causal effect of sugar on hyperactivity [244,245,246,247,248,249,250,251]. In fact, children who are by nature more active seem to need more energy that can be provided by sugar intake [252]. High-carbohydrate intake or general GL do show some positive effects for cognitive processing in adults [253,254]. Just as looking at whole meals can be limiting in understanding how nutrition can support learning, it is important to also focus on specific cognitive processes, like memory and attention. For instance, breakfasts with low GI were associated with better attention [45].

## 6. Conclusions

This review summarizes current knowledge on the effect of diet, physical activity, and sleep on the brain’s cognitive and executive function and children’s ability to learn. Although we did not complete systematic literature reviews or a meta-analysis, we hope that the published research presented here provides an overview of known relationships between modifiable lifestyle factors and children’s ability to learn. We found a relationship between socio-economic factors and parental education status with young children’s attendance in preschools in the US, which indicates that the children who need early exposure to enhanced learning opportunities have limited or no access. This, in combination with the findings that diet quality and the provision of high-quality energy and nutrient sources, opportunities to engage in physical activity, and establishment of good sleep hygiene are important to support learning, indicates a critical need to build supportive environments and community resources to enable children’s optimized learning. Better learning outcomes, in turn, predict better chances to secure work, and develop careers that will lead to improved family environments for future generations. 

We would like to conclude with a cautionary note. Most of the associations and causal relationships discussed in this thematic review are vastly understudied, thus, we do not have sufficient research on critical additional components, interactions between factors (such as mediating or confounding factors), and the higher-level details in the nature of the relationships. Nonetheless, even with the limited knowledge available to date, it is apparent that there is much room for improvement in the US, and globally to build better support systems and grow children’s ability to improve their learning. Overall, changes in local, community, or larger-scale policies and procedures that affect children’s diet, physical activity and sleep patterns should also be viewed through the lens of children’s ability for optimal learning and development, especially for those portions of the population that have very limited resources to compensate for potential shortcomings.
